# Perforated Jejunal Diverticulitis: A Retrospective Observational Study

**DOI:** 10.7759/cureus.97137

**Published:** 2025-11-18

**Authors:** Elena Desmay Hernandez, Nibin Menacherry, Shiva Pratap Unnenahalli Visweswaraiah, Philip Varghese

**Affiliations:** 1 Internal Medicine, Royal Stoke University Hospital, Stoke-on-Trent, GBR; 2 Anesthesiology, Royal Stoke University Hospital, Stoke-on-Trent, GBR; 3 Colorectal Surgery, Royal Stoke University Hospital, Stoke-on-Trent, GBR; 4 Colorectal Surgery, University Hospitals of North Midlands NHS Trust, Stoke-on-Trent, GBR

**Keywords:** abdominal sepsis, acute perforated diverticulitis, complicated diverticulitis, complicated diverticulitis management, jejunal diverticulitis, jejunal perforation, perforated jejunal diverticulitis, retrospective observational study, small bowel diverticulitis

## Abstract

Background: Jejunal diverticulitis is a rare but clinically significant cause of abdominal pain that often mimics other intra-abdominal conditions and carries a risk of serious complications, including perforation. Management strategies have varied between conservative treatment with antibiotics and surgical resection, but evidence has remained limited, particularly in small case series.

Methods: A single-centre, retrospective observational study was conducted at the Royal Stoke University Hospital (UK). Patients diagnosed with jejunal diverticular perforation (JDP) between January 2012 and March 2025 were identified from the electronic patient record system. Data on demographics, presentation, imaging, treatment, complications, and outcomes were collected. Statistical analysis included descriptive summaries and exploratory comparisons between surgical and conservative management groups using Fisher’s exact test, Student’s *t*-test, and Mann-Whitney *U* test. A two-sided *p* < 0.05 was considered significant.

Results: Twelve patients were included (mean age 71.5 ± 14.8 years; 75% male). All had undergone contrast-enhanced CT, which demonstrated jejunal diverticulitis with features of perforation in every case. Seven patients (58.3%) were managed surgically, most commonly with segmental jejunal resection and primary anastomosis, while five patients (41.7%) were treated conservatively with intravenous antibiotics and supportive care. Complications occurred in 42.9% of surgical cases and 40.0% of conservative cases, and no mortality was observed in either group.

Conclusion: Jejunal diverticulitis with perforation was an uncommon but important diagnosis that required early recognition and CT imaging for confirmation. Both conservative and surgical management strategies achieved favourable outcomes, and treatment should be guided by clinical stability, response to antibiotics, and suitability for surgery.

## Introduction

Diverticula are sac-like protrusions that form throughout the digestive tract when the mucosa herniates through the bowel wall, most commonly in the sigmoid colon. Diverticulosis refers to the presence of diverticula without symptoms, while diverticular disease describes symptomatic cases without inflammation or infection, typically presenting with abdominal pain, bloating, or altered bowel habits [1]. The prevalence of diverticular disease increases with age as the intestinal wall weakens, and its pathogenesis is thought to be multifactorial, involving environmental and genetic factors as well as dietary fibre deficiency [1,2]. Major risk factors include diet, obesity, smoking, and the use of non-steroidal anti-inflammatory drugs [2].

In the United Kingdom, diverticulosis is estimated to affect 5% of individuals by the age of 40 and at least 50% by the age of 80 [3]. Many remain unaware of their condition due to its often subtle presentation, such as abdominal pain, constipation, or rectal bleeding. Most cases are managed in primary care with dietary advice, increased fibre, adequate fluid intake, and weight optimisation [4].

While diverticulosis most commonly affects the colon, it is also found in the duodenum (23%) and, less frequently, the jejunum (0.6-4.6%) [5]. Small bowel diverticulosis is believed to result from abnormal motility, raised intraluminal pressures, and possible genetic predisposition [6]. Diverticulitis, occurring in 10-15% of patients with diverticulosis, results from inflammation and micro-perforation [7]. It may be uncomplicated, responding to conservative care, or complicated by abscess, fistula, obstruction, or perforation, which usually requires hospital admission [7].

Jejunal diverticulitis is particularly rare, accounting for 2-6% of diverticulitis cases [5]. It usually affects those over 40 years but may present with non-specific features mimicking appendicitis, colonic diverticulitis, or Crohn’s disease, leading to misdiagnosis or delayed treatment [6]. Complications occur in 30-40% of patients and include stasis with bacterial overgrowth, diarrhoea, malabsorption, pseudo-obstruction, bleeding, recurrent diverticulitis, and, rarely, perforation [8]. Early recognition is therefore crucial.

## Materials and methods

This was a single-centre, retrospective observational study conducted at the large tertiary centre of the Royal Stoke University Hospital (Stoke-on-Trent, United Kingdom). The study included patients diagnosed and treated for jejunal diverticular perforation (JDP) between January 2012 and March 2025. Patients were identified through the Medway CareFlow electronic patient record (EPR) system by searching for diagnostic codes K570 and K571 in any position. Data on patient characteristics and outcomes were extracted from the iPortal EPR system and Medisec letter viewer.

**Figure 1:**
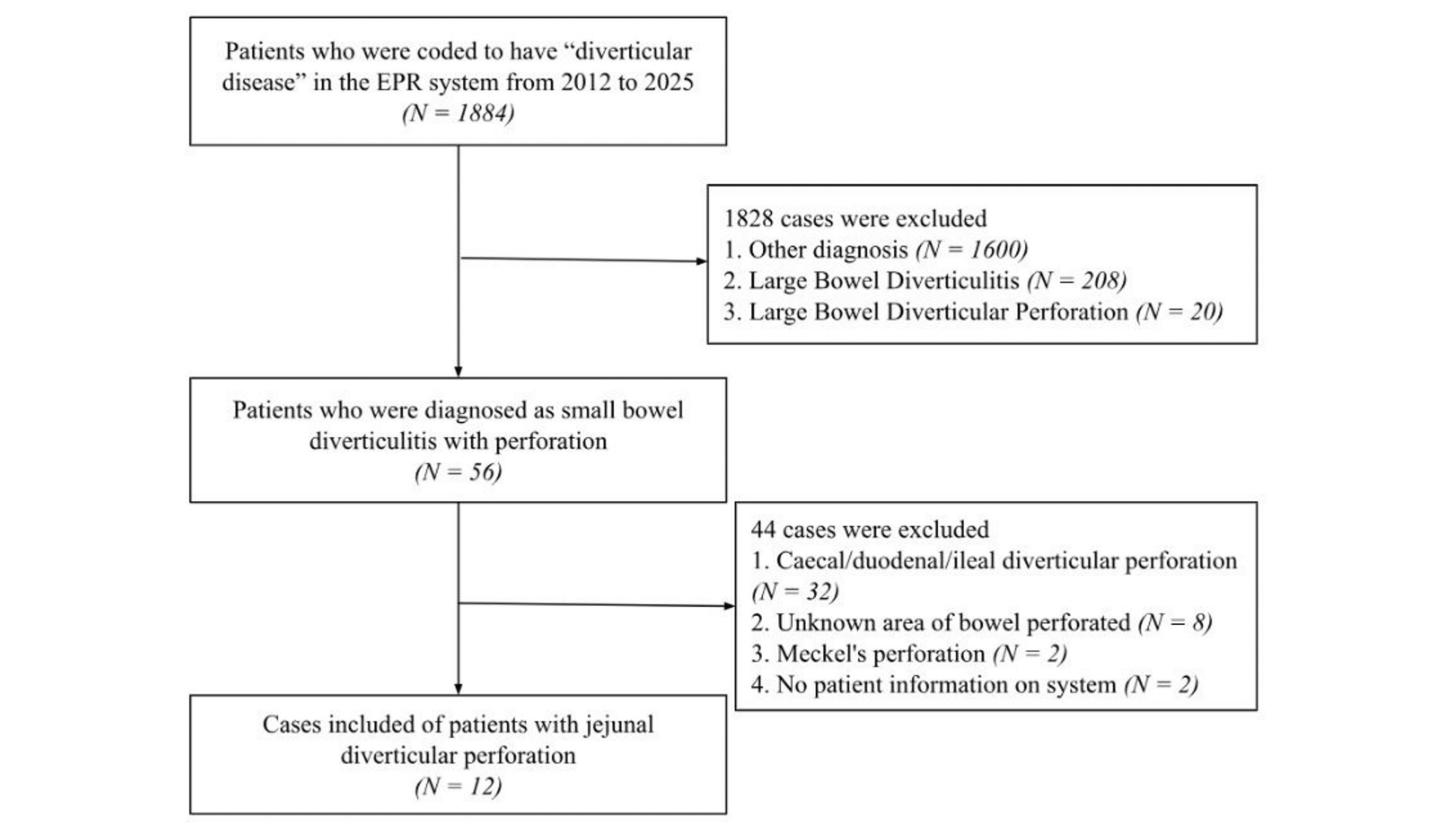
Showing how patients were included from the original data EPR: electronic patient record

The inclusion criteria comprised patients diagnosed with jejunal diverticular perforation for whom complete clinical and radiological records were available in the electronic system. The exclusion criteria included patients with missing or incomplete records, as well as those with other forms of small bowel diverticulitis with perforation not originating in the jejunum, such as cases involving an unidentified segment, Meckel’s diverticulum, the caecum, or the duodenum. Figure 1 illustrates the process by which patients were identified and selected for inclusion in the study according to these criteria.

This study aimed to evaluate the clinical presentation, management strategies, and outcomes of patients with jejunal diverticular perforation at a tertiary care centre, and to compare the results of surgical versus conservative treatment. The outcome of focus for this study was the treatment modalities used for JDP, including the use of antibiotics and surgical methods involved. Secondary outcomes included the incidence of complications and mortality. Baseline characteristics, such as age at treatment, gender, and comorbidities, were recorded. Additionally, data were collected on the initial diagnosis, imaging findings, clinical presentation, treatments administered (including antibiotics), complications, and outcomes. Surgical intervention was defined as any procedure requiring theatre admission to seal the perforation, while conservative management was defined as non-surgical treatment, including intravenous fluids, antibiotics, and placement of enteral tubes.

This retrospective study was conducted in accordance with the principles of the Declaration of Helsinki. Institutional approval was obtained from the University Hospitals of North Midlands NHS Trust Clinical Audit and Effectiveness Department (Reference no. CA15225). As the study involved anonymised retrospective data and no direct patient contact, the requirement for informed consent was waived.

Statistical analysis

Analyses were performed using standard statistical software (e.g., R v4.x (R Foundation for Statistical Computing, Vienna, Austria) or SPSS v2x (IBM Corp., Armonk, NY, USA)). Continuous variables are presented as mean ± standard deviation (SD) when approximately normally distributed, and median (interquartile range, IQR) when skewed. Categorical variables are summarised as N (%). Given the small sample, comparisons between conservative and surgical groups were considered exploratory: Fisher’s exact test was used for categorical data; Student’s t-test or Mann-Whitney U was used for continuous data as appropriate after visual inspection and, where relevant, Shapiro-Wilk testing. Effect sizes (e.g., risk difference or odds ratio) are reported with 95% confidence intervals when informative. Two-sided p < 0.05 was taken to indicate statistical significance. No imputation was performed; analyses used available data only. The sample size precluded reliable multivariable modelling.

## Results

Baseline characteristics

Twelve patients were included, seven (58.3%) treated surgically and five (41.7%) managed conservatively. Baseline demographics are shown in Table 1. The groups were similar in age and sex, with no statistically significant differences.

**Table 1:** Baseline Characteristics Values are mean ± SD or N (%). p-values from Student’s t-test and Mann–Whitney U (MWU) for continuous variables, Fisher’s exact test for categorical variables. Two-sided p < 0.05 considered significant.

Characteristic	Surgical (N = 7)	Conservative (N = 5)	P-value
Age, Mean ± SD (minimum-maximum)	70.7 ± 12.0 (85 - 48)	72.6 ± 20.9 (89 - 36)	0.86 (T-test)
Sex: Male, N (%)	6 (85.7)	3 (60)	0.52 (Fisher)
Sex: Female, N (%)	1 (14.3)	2 (40)	0.52 (Fisher)

Clinical presentation and radiology

All patients presented with abdominal pain, nausea, vomiting, and generalised tenderness; most had signs of peritonitis. Contrast-enhanced CT was performed in all cases and was instrumental in diagnosis. The most common findings were jejunal diverticulitis with contained perforation, fat stranding, extraluminal gas, and small collections. In a minority, the exact site of perforation was only confirmed intra-operatively.

Antibiotic usage

All patients (100%) were commenced on antibiotics at admission, in line with University Hospitals of North Midlands (UHNM) guidelines. Co-amoxiclav was most commonly prescribed, while aztreonam + metronidazole + vancomycin were used in those with penicillin allergy. Broad-spectrum agents (Tazocin or meropenem) were reserved for patients with sepsis or failure of first-line therapy. There were no significant differences in prescribing patterns between groups (Table 2).

**Table 2:** Antibiotic usage by management group Values are N (%) with exact 95% confidence intervals (CI). p-values from Fisher’s exact test. Two-sided p < 0.05 considered significant.

Characteristic	Surgical (N = 7)	Conservative (N = 5)	P- Value
Co-amoxiclav	3 (42.9)	3 (80)	1
Aztreoman + Metronidazole + Vancomycin	1 (14.3)	1 (20.0)	1
Tazocin	2 (28.6)	0 (0.0)	0.47
Meropenem	1 (14.3)	0 (0.0)	1

Surgical management

Seven patients (58.3%) required surgical intervention. The main procedure performed was segmental jejunal resection with primary end-to-end anastomosis, undertaken for patients with clinical peritonitis, sepsis, or failure of conservative therapy. In selected cases, adhesiolysis or abdominal washout was carried out in addition to resection. Operative findings typically revealed perforated diverticula with surrounding inflammation, localised collections, and adhesions. The surgical approach was predominantly via laparotomy, and anastomoses were created using stapling devices. Postoperative outcomes were favourable overall, with only short-term complications such as collections and wound dehiscence observed.

Outcomes

Overall, five of 12 patients (41.7%) developed complications as shown in Table 3. Rates were similar between the surgical and conservative groups (42.9% vs 40.0%, Fisher’s exact test p = 1.00). In the conservative group, complications included recurrent diverticulitis and collections requiring drainage. Among surgically treated patients, complications included postoperative collections, wound dehiscence, and recurrent diverticular symptoms. Four surgically treated patients (57.1%) and three conservatively treated patients (60.0%) had no subsequent complications. No deaths were observed (0/12, 0%).

**Table 3:** Outcomes by management group Values are N (%) with exact 95% confidence intervals (CI). p-values from Fisher’s exact test. RD = risk difference. Two-sided p < 0.05 considered significant.

Characteristic	Surgical (N = 7)	Conservative (N = 5)	P- Value / Effect size
Complications	3 (42.9) [95% CI 9.9–81.6]	2 (40) [95% CI 5.3–85.3]	1.00 (Fisher); RD 2.9% (95% CI –53.6 to 59.3)
Mortality	0 (0) [95% CI 0–41.0]	0 (0) [95% CI 0–52.2]	NA (no events)

## Discussion

This retrospective series represents one of the few reports of JDP from a UK tertiary centre. The demographic profile of our patients - predominantly older adults with comorbidities, though occasionally younger individuals - reflects that described in the literature. Krishnamurthy et al. reported that small bowel diverticulosis predominantly affects those over 60 years, with jejunal diverticula accounting for around 80% of small bowel cases [9]. Similarly, Kassahun et al. highlighted the typical late presentation in elderly patients, often with delayed diagnosis due to non-specific symptoms [10]. Our cohort, which included both older and younger patients, reinforces that JDP, while rare, should remain a differential diagnosis across a broad age range.

Diagnosis and imaging

All patients underwent contrast-enhanced CT of the abdomen and pelvis, which was central to diagnosis. CT findings in our series - fat stranding, mesenteric oedema, extraluminal gas, and localised collections - are in line with previous studies where CT was found to be the most sensitive modality for detecting small bowel diverticulitis and its complications [11]. In some patients, however, the precise site of perforation was confirmed only intra-operatively, echoing reports that CT, while sensitive, can occasionally fail to localise perforations due to overlapping radiological features with other pathologies [11].

Antibiotic therapy

Every patient received antibiotics, with prescribing patterns reflecting local UHNM guidelines. Co-amoxiclav was the most frequent first-line therapy, with aztreonam-based regimens used for penicillin allergy and escalation to broad-spectrum therapy reserved for those with sepsis or inadequate response. This mirrors international recommendations, including NICE guidance for complicated diverticulitis, which emphasises tailoring antimicrobial therapy to allergy status, clinical severity, and local microbiological resistance patterns [4]. Previous series also describe high reliance on broad-spectrum regimens in complicated small bowel diverticulitis, underscoring the importance of early intravenous therapy [6].

Surgical versus conservative management

Management in our series was nearly evenly split between conservative and surgical strategies. Conservative treatment, including intravenous antibiotics and bowel rest, was reserved for stable patients or those unfit for surgery, while surgical resection with primary anastomosis was performed for peritonitis, sepsis, or worsening clinical status. Kassahun et al. reported that conservative therapy can be effective for uncomplicated cases but carries the risk of recurrence, while surgery offers definitive treatment in complicated diverticulitis [10]. Other series similarly note that laparotomy with segmental resection and primary anastomosis is the preferred surgical option, with resection margins guided by the extent of diverticulosis and tissue viability [12].

Outcomes

In our study, complication rates were similar between groups (42.9% surgical vs 40.0% conservative), consistent with other small case series that show no clear superiority of one approach when patients are appropriately selected [13]. Conservative complications included recurrence and persistent collections requiring drainage, while surgical complications involved postoperative collections, wound dehiscence, and recurrent diverticular symptoms. Importantly, there were no deaths in our cohort. Historical mortality rates for perforated jejunal diverticula are significantly higher, ranging from 24% following surgery to up to 40% overall in perforated cases [12]. Kassahun et al. and Harbi et al. both emphasise that early CT-based diagnosis and prompt intervention are crucial in reducing mortality [10,11]. Our findings support this view, with improved outcomes likely attributable to early imaging, timely antibiotics, and tailored peri-operative care.

Interpretation

Taken together, these findings reinforce that both conservative and surgical management are valid strategies for JDP. Conservative therapy is appropriate for stable or high-risk patients responding to antibiotics, while surgery remains the treatment of choice for those with peritonitis, clinical deterioration, or non-response to medical therapy. Our series supports prior reports suggesting that surgery may reduce recurrence in the long term, though both approaches can be associated with good outcomes when selected appropriately.

Limitations

This study has several limitations. First, the retrospective observational design makes it prone to information bias, as data were extracted from electronic records and clinical letters, which may not consistently capture all relevant details. Second, the small sample size of 12 patients limits the power of statistical comparisons and precludes reliable multivariable analysis. The single-centre nature of the study also restricts generalisability, as the findings reflect the experience of one tertiary hospital and may not represent wider clinical practice. Third, the search strategy, based mainly on diverticular disease coding, may have excluded cases recorded under alternative small bowel diagnoses. In addition, treatment decisions were clinician-driven rather than protocolised, introducing potential selection bias when comparing conservative and surgical approaches. Further methodological constraints included variable documentation quality, limited follow-up data, and the absence of standardised diagnostic and outcome definitions. Despite these limitations, this study provides valuable real-world insight into the presentation and management of a rare condition.

## Conclusions

Jejunal diverticulitis with perforation is a rare but important cause of generalised abdominal pain, requiring a high index of suspicion. Although most commonly affecting older patients, it may also occur in younger individuals. Contrast-enhanced CT scanning provides the most reliable means of diagnosis. Management should be tailored to the clinical picture: stable or high-risk patients may be managed conservatively with fluids, bowel rest and antibiotics, while those with peritonitis or failure of medical therapy are appropriate for surgical resection with primary anastomosis. Both approaches yielded favourable outcomes in our series, and choice of strategy should be guided by patient stability, response to antibiotics and surgical fitness.
